# Towards a lifelong personalized brain health program: empowering individuals to define, pursue, and monitor meaningful outcomes

**DOI:** 10.3389/fneur.2024.1387206

**Published:** 2024-06-05

**Authors:** Stina Saunders, Joyce Gomes-Osman, Ali Jannati, Marissa Ciesla, Russell Banks, John Showalter, Graciela Muniz-Terrera, Saturnino Luz, Craig Ritchie, Álvaro Pascual-Leone

**Affiliations:** ^1^Linus Health Inc., Boston, MA, United States; ^2^Usher Institute, University of Edinburgh, Edinburgh, United Kingdom; ^3^Department of Neurology, University of Miami Miller School of Medicine, Miami, FL, United States; ^4^Department of Neurology, Harvard Medical School, Boston, MA, United States; ^5^Department of Communicative Sciences & Disorders, College of Arts & Sciences, Michigan State University, East Lansing, MI, United States; ^6^Ohio University Heritage College of Osteopathic Medicine, Ohio University, Athens, OH, United States; ^7^Scottish Brain Sciences, Edinburgh, United Kingdom; ^8^Population and Behavioural Sciences Division, University of St Andrews, St Andrews, United Kingdom; ^9^Marcus Institute for Aging Research and Wolk Center for Memory Health, Hebrew SeniorLife, Boston, MA, United States

**Keywords:** patient reported outcomes, clinical meaningfulness, Alzheimer’s disease, mild cognitive impairment, outcome measures

## Abstract

Incorporating person-centered outcomes into clinical trials for neurodegenerative diseases has been challenging due to a deficiency in quantitative measures. Meanwhile, the integration of personally meaningful treatment targets in clinical practice remains qualitative, failing to truly inform evaluations, therapeutic interventions and longitudinal monitoring and support. We discuss the current advances and future directions in capturing individualized brain health outcomes and present an approach to integrate person-centered outcome in a scalable manner. Our approach stems from the evidence-based electronic Person-Specific Outcome Measure (ePSOM) program which prompts an individual to define personally meaningful treatment priorities and report level of confidence in managing items that matter to the individual the most (e.g., “Do I feel confident in my ability to contribute to a conversation?”). Deployed either as a single version (person only) or a dyad version (person and care partner), our proposed tool could be used as an endpoint in clinical trials, offering proof of meaningful intervention benefits and in clinical practice, by establishing an anchor for the therapeutic objectives sought by the individual.

## 1 Introduction

The recognition that measuring treatment benefits in medicine needs to have a personally meaningful focus may reach as far back as the beginning of modern medicine. Greek medicine demonstrated what we still see as true – diagnostic reasoning starts with medical history, and medical history needs to be focused on the person. As posed by Centor (1): Who is this patient? What are the patient’s goals? How might the patient’s personal situation impact our treatment options? Or as articulated by Sir William Osler: “The good physician treats the disease; the great physician treats the patient who has the disease” (1). To practice great medicine, incorporating the patient’s voice and personal treatment priorities is critical.

**Figure 1:**
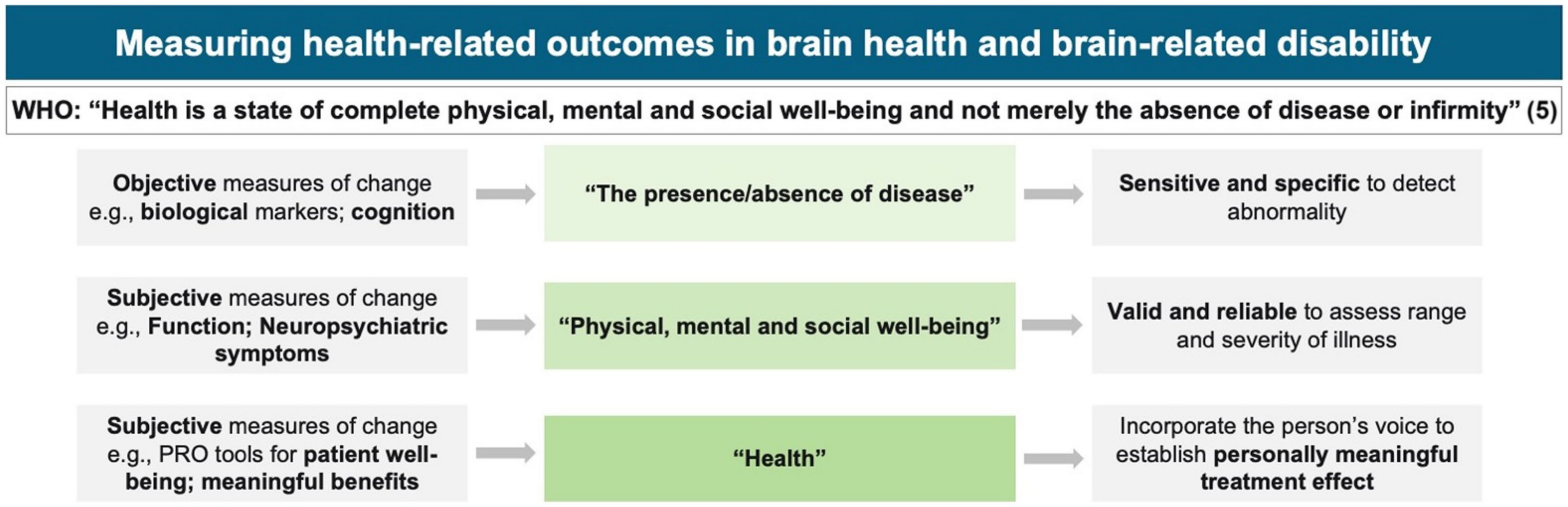
Health-related outcomes in Alzheimer’s disease: an intervention should demonstrate a personally meaningful impact and benefits (5).

To this end, models such as value-based healthcare (2) or realistic medicine (3) have been proposed to promote personally defined treatment priorities. These models align with the International Classification of Functioning, Disability, and Health, which has emphasized the need for personalization for 20 years (4), yet despite this long-standing awareness and funding for numerous initiatives such as the Patient-Centered Outcomes Research Institute in the United States or the International Consortium for Health Outcomes Measurement, significant challenges remain in incorporating the individual’s voice, particularly in the brain health field (Figure 1). At the same time, for neurodegenerative diseases like Alzheimer’s disease (AD) and related dementias, regulatory bodies that approve new medications emphasize the need to establish an intervention’s meaningfulness on an individual patient’s function and well-being, aside from the effect on underlying disease pathology (6, 7).

This perspective paper explores the challenges and highlights the developments and opportunities in integrating metrics related to personally meaningful outcomes in brain health and the care of individuals with neurodegenerative diseases and their families. We outline an approach to define, monitor, and protect brain health priorities that matter the most to an individual, and propose a method for how both clinical trials of new treatments and interventions, as well as healthcare delivery, can truly be guided by and cater to person-centered priorities, enable prevention, and encourage taking action rather than reacting (8).

### 1.1 Path to person-centered outcomes in research

The challenges of capturing person specific subjective information are distinctly different in clinical research and clinical practice. The main challenge in clinical research is that therapeutics specifically target objectively quantified pathologies but should really impact a patient’s function and well-being to demonstrate clinically meaningful benefits. Consider for example the recent approvals by the US Food and Drug Administration (FDA) for two disease-modifying therapies for AD: aducanumab (Aduhelm®) (9) and lecanemab (Leqembi®) (10). Both of these agents were shown to significantly reduce the amyloid brain burden. However, demonstration of clinical impact was much more elusive, arguably with only lecanemab showing beneficial effects - as compared with placebo - in slowing down the trajectory of the patient’s cognitive decline, as demonstrated for example by performance in everyday activities. Whether or to what degree such clinical meaningfulness was associated with the amyloid-clearing mechanism of action remains unclear. Similarly, it is unclear how clinically meaningful effects at the group level translate to effects at the individual patient level. Regardless, this marks a significant change from prior trials of anti-amyloid monoclonal antibodies that showed a reduction of amyloid load but no observed clinical or cognitive benefit (11). From this standpoint, we highlight that regulatory bodies, clinicians, individuals, and families making decisions about the risks and benefits of a novel therapeutic intervention require information specifically about the functional, clinically meaningful benefits of the treatment.

Clinical meaningfulness is hard to define, quantify, and broadly implement in clinical research for several reasons. Suitable outcome measures need to have appropriate psychometric properties to qualify for clinical trial inclusion and be both scalable and pragmatic to incorporate into study protocols. Importantly, clinical meaningfulness is ultimately uniquely personal and thus subjective. Assessing the meaningful effect of a given treatment on a given person can only be achieved through a person-centered approach, using tools such as patient-reported outcome (PRO) measures. Broad acceptance of such metrics requires PRO measures to be standardized to fulfill regulations in different countries, yet they will also need to be sufficiently specific to capture the aspects of “functioning” that are unique to each ethnicity, culture, nation, and population.

Outcome measures currently used in AD trials assess interventions’ benefits through group-level comparisons using tools that do not account for individual treatment targets. For example, our previous work suggests that items like ‘Using technology’ and ‘Following a storyline in a movie’ are commonly considered important outcomes for older adults (12), but this level of granularity would not be captured in a list of a limited number of pre-defined items derived as the ‘average’ person from population data. Additionally, our previous work demonstrates that there are differences in what individuals define as personally meaningful depending on their sociodemographic backgrounds. For instance, we found that individuals with higher education were significantly more likely to report certain items like ‘Discussing literature and science’ and ‘Playing musical instruments’ as important treatment outcomes compared to individuals with lower education (12). A PRO tool would therefore ideally capture meaningful change individually *and* at a group level.

Whilst there are dementia-specific PRO tools that capture the four health-related quality of life domains established by the Centers for Disease Control and Prevention (symptom, physical function, psychological well-being, and social functioning) (13), such as quality of life measures like EQ-5D (14), DEMQOL (15) or Quality of Life in Alzheimer’s Disease (QoL-AD) (16), and ADL measures such as the Alzheimer Disease Cooperative Study Activities of Daily Living Scale (ADCS-ADL) (17), these tools may fail to capture specific areas that are personally meaningful to individuals. Furthermore, they often require a proxy to be completed, and there may be discrepancies between the self- and observer-reported results (18). What is more, these measures have typically been developed for use in patients with dementia but there may be significant differences in the things that matter to the individual along the disease spectrum in terms of what “good” brain health looks like (12), making many of these outcome measures not applicable for use at preclinical or prodromal stages of Alzheimer’s disease and related dementias (ADRD).

One of the tools recently named by the FDA as an example of a possible solution for including the individual’s voice in measuring treatment outcomes is the Goal Attainment Scale (GAS) (19). However, the GAS was originally developed in the brain injury and rehabilitation setting and thus may not be ideally suited to neurodegenerative processes. Furthermore, there are considerations around the psychometric properties of the GAS (20) due to its non-standardized nature as well as practical limitations like the requirement of a clinician to administer the tool and the length of time the administration takes (21).

Some of the core features of the above-mentioned PRO measures pose a significant challenge to the wider adoption of these types of tools e.g., the availability of a clinician to administer the tool, need for a reliable proxy and challenges around quantifying output from these tools. A PRO tool needs to have robust psychometric properties, but it needs to also be practical and feasible to administer. We present an overview of the required PRO qualities in research and healthcare settings in Table 1.

**Table 1:** Critical considerations for PRO instruments in research and clinical practice.

PRO tool criteria	Why is it important?	Research	Clinical practice	Relevance
Validity	The core of a PRO tool is to measure the “right” content, i.e., the tool measures the most important aspects of a person’s brain health.	The tool should account for individual differences in brain health priorities. To qualify as a valid and applicable PRO tool, the ePSOM programme was developed with a person-specific focus (self-defined “what matters most”).	The tool needs to help the clinician better understand what matters uniquely to an individual patient, i.e., what the patient fears losing and what the patient wants to maintain the most.	The tool needs to meet robust psychometric properties to be used as a clinical trial endpoint and have high clinical utility in clinical settings.
Reliability	It is critical that the tool yields consistent results for the same participant over time.	The tool should allow for effective comparisons across studies with reliable observed effects. The ePSOM tool’s psychometric properties are currently being established in longitudinal validation studies.	To obtain accurate and dependable results, a PRO tool should have clear instructions and be easily understood by patients. The test–retest reliability should ensure that if a patient’s condition remains stable, their responses to the PRO tool remain consistent over time.	The tool needs to provide consistent and reliable information in routine care for use in clinical settings.
Interpretability	The output of the tool needs to be easily understood, meaningful, and relevant.	Output from a PRO tool in ADRD could be related to objective study outcomes as further proof of an intervention’s effectiveness that incorporates the participants’ voice. The ePSOM tool’s population-level and ADRD-specific normative data are currently being established in validation studies.	A person-centered approach makes utilizing a PRO tool meaningful and relevant. The ePSOM tool yields a score indicating high or low confidence in being able to do the things that matter the most to an individual with color-coded categories that can be easily communicated to the individual.	This is important in research to be able to relate changes in PRO scores to other measures or benchmark tools. It is important that a PRO tool is easy to interpret and that the scores reflect the patient’s true status.
Assay Responsiveness	In ADRD, to understand if a treatment is effective, the tool needs to reliably detect decline (in the placebo group) and stability (in the treatment group).	To be able to capture Minimal Important Difference, the tool should be able to capture treatment effects if an intervention is effective. To achieve high sensitivity, the scale of the ePSOM tool is constructed around a granular assessment of decline (vs. stability) to detect a treatment effect if one exists.	A person-centered PRO tool should help the clinician tailor follow-up care that meets the unique needs of an individual patient, i.e., establish a need for further treatment or change the clinical management. The tool should also be able to reflect the therapeutic effect, to the extent that a treatment is effective in addressing those unique needs.	In research, the tool needs to capture changes in brain health in a way that is relevant to the treatment’s intended effects. The tool needs to be sensitive to changes in a patient’s underlying disease.
Sensitivity to Change	The tool needs to detect meaningful changes in individuals’ brain health status over time.	To detect change attributable to treatment effect or unrelated to treatment effect, a PRO tool needs to exhibit sensitivity to measuring treatment effects in clinical trials longitudinally. The ePSOM tool’s responsiveness is currently being established in longitudinal validation studies.	A PRO tool should help ascertain if an intervention or treatment leads to any changes in brain health in routine clinical practice.	It is critical to incorporate the person’s point of view in measuring change over time in both settings.
Feasibility	A PRO tool needs to be practical to administer and easily understandable by a diverse group of patients including use of appropriate literacy levels.	To fulfill the feasibility criteria, the ePSOM tool has been developed under ISO 13485 certified product development process. This guarantees the tool’s feasibility in research settings, ensuring accessibility, user-friendliness, and adherence to the highest medical device standards.	The ePSOM tool is compliant with regulatory standards, designed to be fully digital for ease-of-application, accessible to patients with various literacy levels, with remote administration and self-report.	There is greater time pressure, and a need for efficiency and feasibility in the clinical setting whereas researcher can allocate more time for completion of PRO tools.
Socio- demographic and cultural standard	A PRO tool needs to avoid measurement bias so the tool is equally relevant and useful across all socio-demographic groups.	The ePSOM framework ensures the tool is culturally and linguistically appropriate for diverse populations.	The administration of the questionnaire improves doctor-patient communication because the tool helps elicitate what matters uniquely to this patient across all socio- demographic and cultural backgrounds.	The tool needs to be applicable, understandable and relevant across all socio-demographic and cultural backgrounds in both settings.

### 1.2 Path to person-centered outcomes in clinical practice

In clinical practice, a PRO tool must capture the breadth and depth of the information shared during a meaningful clinical encounter. One important challenge to overcome is clinicians’ skepticism about whether standardized instruments can provide any added value in eliciting information about their patients when clinicians do thorough examinations already, especially in populations with diverse sociodemographic backgrounds (22). A critical component in the clinical deployment of PROs is capturing outcomes on a personalized rather than group level. Additional considerations for broad implementation are healthcare systems needing to decide how the PRO data would be managed and used for clinical purposes as well as having resources available to train clinicians (23).

From the clinician’s perspective, capturing information about who the patient is, their priorities, and their personal situations is crucial as it can impact treatment options, but these preferences should be incorporated into the workflow without adding additional documentation burden. Having reliable PROs can help efficiently guide the clinician/patient conversation to clarify the desires of the patients. Essentially, a measure must be developed, designed, and implemented to enable clinicians to do what they already do easier and better. Furthermore, the rational in capturing such patient-centered metrics has to be to guide interventions and inform serial assessments to monitor patients’ progress and identify appropriate timing for further evaluations or treatment modifications.

The information in the medical history can provide as much as 75% of the information necessary to make an accurate diagnosis (24), even before a physical exam and diagnostic tests. Importantly, “feeling listened to” is critical for the patient-physician relationship, where a provider’s failure to listen can lead to the patient’s disengagement and feeling helpless (25–27). Although clinicians may subjectively ask about personal treatment priorities, there are no quantitative outcome measures to monitor treatment success against or compare different populations. Therefore, we emphasize that individuals receiving healthcare must be appropriately empowered to define their individual priorities as treatment outcomes.

A review of PRO tools in healthcare settings concluded that clinicians considered individualized PRO tools to enable patients to “tell their story” in their own words and provide the clinicians an opportunity to check that they understood the individual’s intended meanings (28). Furthermore, the use of PRO tools has been demonstrated to offer satisfaction both to the clinician and the individual, as well as improve communication. For example, an oncology study concluded that an individual’s well-being, as captured by PROs, was more likely to be discussed when the clinical team received output from the PRO tool and clinicians could examine the impact of interventions on PRO scores (29).

Finally, we note that in the advanced stages of brain disease when dementia manifests, individuals will still have personally meaningful things that are unique to them. These preferences should become objectives and metrics that need to be considered by both the care partner and the clinical team. For instance, if individuals’ wishes are not recorded in advanced care directives or healthcare proxy documents while the person still has the capacity to express the outcomes that matter to them, the person may be unable to articulate these personally meaningful areas later in the disease stage.

We argue that a PRO tool with remote, at-home administration capabilities that addresses individualized, person-centered health domains and yields a quantitative score would allow individuals time to explain what matters to them and help maximize the efficiency of study visits or in-clinic visits with healthcare providers.

## 2 Discussion

**Figure 2:**
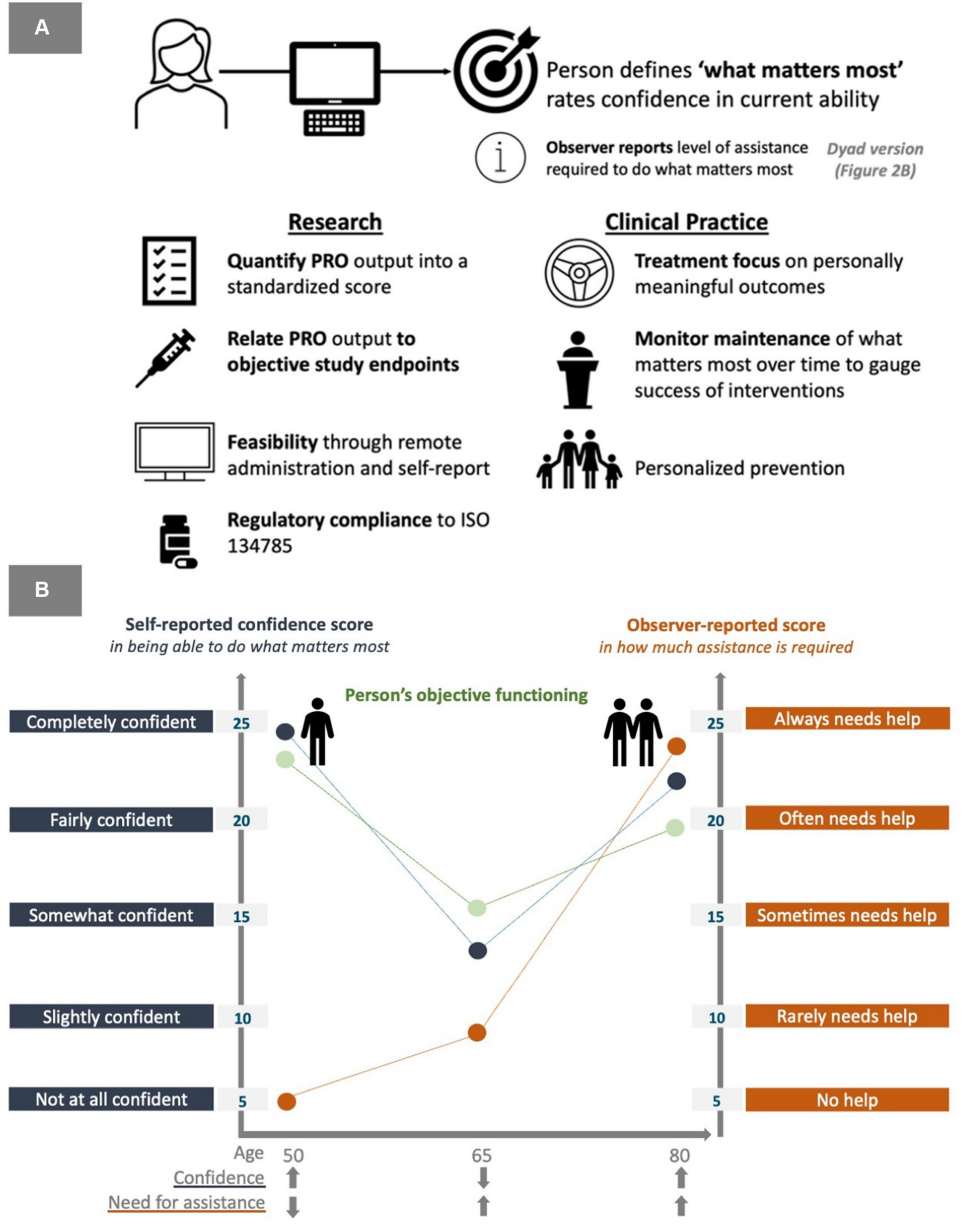
**(A)** The ePSOM approach to personalize outcomes in research and clinical practice. **(B)** A model of self-reported ability to do the things that matter the most in relation to observer-reported dependency to achieve the things that matter the most.

We recognize that the future of healthcare lies in personalized medicine (30). As such, we propose an approach to integrate technology into the brain health field to both monitor and support outcomes that truly matter to patients in the long term. It seems critical that new interventions incorporate a person-centered approach and a digital solution designed to generate individualized feedback would empower patients in healthcare delivery (Figure 2). Technological solutions offer an opportunity to reflect an intervention’s clinical effectiveness from the point of view of the research participant and incorporate input about individual treatment priorities in healthcare delivery. What is more, technological platforms promise to enable the development of scalable, reliable and valid solutions.

Building on research by Saunders et al. (31), we highlight the electronic Person Specific Outcome Measure (ePSOM) as a potential tool in this space. In our approach, we argue that what constitutes “meaningful treatment benefits” for individuals living with brain health impairment should be determined at an individual rather than group level. The ePSOM approach, now licensed to and developed by Linus Health, incorporates unique input from the individual and care partner, with a scale that quantifies the severity of the disability based on self-reported confidence in being able to do the things that matter the most to the individual. Additionally, a dyad version of the tool incorporates an observer-reported level of assistance required to manage the unique priority outcomes, offering insights into subjective well-being and objective assessment of dependence. We note that the person-centered framework could also be applied to capturing care-partner burden that is personalized to each care partner completing the assessment.

However, it is unlikely that the same PRO solution would fit into the distinct workflows of both clinical practice and clinical research. For example, workflows in healthcare, especially primary care, are among the most time-pressured, so PRO solutions need to be substantially briefer than in clinical research. A key clinical utility of using person-specific tools in healthcare settings is forming the foundation upon which care management, assessment of cognition and function (e.g., motor skills, physical performance), and interventions should be based.

Whilst in a research setting, the critical aspect of a PRO tool is monitoring change over time with good test–retest reliability, and quantitative features that allow to assess whether and by how much a given intervention induces a change, in a clinical setting a PRO tool should facilitate actionable and personally relevant recommendations an individual can put into practice rather than merely quantify change over time. This is evidenced by an increasing number of Brain Health Clinics producing tangible “take home” outputs to hand to the person.

In our approach in the healthcare setting, we consider the individualized patient-reported input, along with other brain health factors and lifestyle questions in the generation of a personalized Brain Health Action Plan, a patient education tool that highlights areas of attention and actionable lifestyle and health interventions that an individual can take toward better brain health. This type of multimodal brain health program simultaneously addresses 8 pillars of brain health: (1) Build your village; (2) Stay true to your purpose; (3) Be mindful about your mental health; (4) Keep your health in check; (5) Eat lean and green; (6) Move every day; (7) Get good rest; and (8) Learn something new. Responses to the lifestyle questions identify the brain health pillars where individuals are doing well and should continue the good work, as well as brain health pillars that need more attention. Importantly, personalized outputs like the Brain Health Action plan link what matters most to the person with actionable lifestyle and health interventions that are tailored to the person’s needs. Incorporating individualized input from the person allows the identification of specific skills and actions that can be taken to maintain function in the individual’s personally meaningful areas, as well as most relevant assessments to monitor function in these priority areas. The personalized workflow is an iterative process, integrating learnings gained from specific interventions, improving specific outcomes into future personalized healthcare, and thus offering reliable metrics for assessing the efficacy of therapeutic interventions and longitudinal evaluation of individual disease trajectories.

## 3 Conclusion

The current paper discusses the importance and the challenges of capturing patient-centered outcomes in research and clinical practice, and outlines a practical and effective approach to define, monitor, and protect person-centered brain health priorities.

The workflow of using personally defined treatment priorities as benchmarks to evaluate treatment efficacy against, anchors all clinical decision-making around the desired outcomes as treatment targets. In this approach, person-specific metrics arise both from subjectively reported well-being such as self-reported confidence in being able to do the things that matter the most, as well as objective measures of change which are mapped to individually defined treatment targets. Adding the care partner’s perspective offers a more nuanced assessment of functioning and a transition from independence to reliance, whilst placing the individuals’ wishes at the center of clinical decision-making.

Brain health-related illnesses impact an individual’s functioning through a range of pathways that are disease-, personality-, and culturally specific and therefore are unique to each person. Ultimately, disease modification to improve brain health, be it through pharmaceutical or lifestyle intervention, is only relevant in terms of the intervention’s impact on the disease (biomarker) and impact on the person (PRO) – all other assessments are surrogate metrics for brain health.

## Data availability statement

The original contributions presented in the study are included in the article/supplementary materials, further inquiries can be directed to the corresponding author.

## Author contributions

SS: Conceptualization, Methodology, Project administration, Visualization, Writing – original draft, Writing – review & editing. JG-O: Conceptualization, Writing – review & editing. AJ: Conceptualization, Writing – review & editing. MC: Writing – review & editing. RB: Writing – review & editing. JS: Writing – review & editing. GM-T: Writing – review & editing. SL: Writing – review & editing. CR: Writing – review & editing. ÁP-L: Conceptualization, Supervision, Visualization, Writing – review & editing.
